# Screening data for the endocrine disrupting activities of 583 chemicals using the yeast two-hybrid assay

**DOI:** 10.1016/j.dib.2018.11.071

**Published:** 2018-11-23

**Authors:** Fujio Shiraishi, Ryo Kamata, Masanori Terasaki, Hidetaka Takigami, Yoshitaka Imaizumi, Mayuko Yagishita, Daisuke Nakajima

**Affiliations:** a National Institute for Environmental Studies, 16-2 Onogawa, Tsukuba, Ibaraki 305-8506, Japan; b Kitasato University, 23-35-1 Higashi, Towada, Aomori 034-8628, Japan; c Iwate University, 3-18-8 Ueda, Morioka, Iwate 020-8550, Japan

## Abstract

We screened 583 chemicals for receptor binding activity to the human estrogen receptor (hER), the Japanese medaka estrogen receptor (medER), and the aryl hydrocarbon receptor (AhR) using the yeast two-hybrid assay. The substances tested included substances that could potentially be produced unintentionally by industrial processes, such as halogenated steroids and phenols. Antagonistic effects on hER and the androgen receptor were also screened. The test chemicals were selected for screening on the basis of chemical structure associated with possible estrogen receptor binding activity. The current study presents the report on the screening of 583 chemicals for different kinds of endocrine disrupting activity.

**Specifications table**

**Table:** 

Subject area	Biology
More specific subject area	Toxicology
Type of data	Table
How data was acquired	Yeast two-hybrid assay. Luminescence was read on a 96-well plate luminometer (Luminescencer JNR AB2100; Atto Corp., Tokyo, Japan)
Data format	Raw data
Experimental factors	583 chemicals of receptor binding activities, yeast toxicities, and photobacterium toxicities were tested. Each positive control was 17β-estradiol (hER-, medER-agonistic, β-naphthoflavone (AhR-agonistic)), 4-hydroxy-tamoxygen (ER-antagonistic), and 1-nitropyrene (AR-antagonistic).
Experimental features	The hERα-, medERα-, and AhR-agonist activities and hERα- and AR-antagonist activities of the 583 test chemicals were measured using a yeast two-hybrid assay system. The hERα, medERα, or human AhR and the coactivator TIF2 were introduced into each yeast cell (Saccharomyces cerevisiae strain Y190) in accordance with the method by Nishikawa [1].
Data source location	Tsukuba, Ibaraki, Japan
Data accessibility	All data are presented in this article.
Related research article	Kamata R., Shiraishi F., Nishikawa J., Yonemoto J. and Shiraishi H., 2008. Screening and detection of the in vitro agonistic activity of xenobiotics on the retinoic acid receptor. Toxicol. in vitro. 22, 1050–1061 [2].
	Kamata R., Nakajima D., and Shiraishi F., 2018. Agonistic effects of diverse xenobiotics on the constitutive androstane receptor as detected in a recombinant yeast-cell assay. Toxicol. in vitro. 46, 335–349 [3].

**Value of the data**

• Screening for endocrine disrupting activity of 583 chemicals was conducted by yeast-two hybrid assay.
• These data are evaluated; because few studies have carried out to evaluate several kinds of endocrine disrupting activities for same assay system and same timing.
• Each endocrine disrupting activity of 583 chemicals are shown with not only positive or negative, but also each activity values.
• This is a first report on estrogen receptor binding activities of Japanese medaka.
• Several synthesis chemicals which may be released as unknown chemicals with endocrine disrupting properties by accident are included in 583 chemicals.

## 1 Data

The substances tested included substances that could potentially be produced unintentionally by industrial processes, such as halogenated steroids and phenols. Antagonistic effects on hER and the androgen receptor were also screened. The test chemicals were selected for screening on the basis of chemical structure associated with possible estrogen receptor binding activity. The main positive chemical groups associated with each receptor binding activity were endogenous hormones (for hER or medER), and polycyclic aromatic compounds (AhR), respectively, see Table.

## 2 Experimental design, materials, and methods

hERα, medERα, CAR, and AhR agonist activities and hERα and AR antagonist activities of the 583 test chemicals were measured with a yeast two-hybrid assay system. Introduced into each yeast cells (Saccharomyces cerevisiae Y190) were the human estrogen receptor (hERα), Japanese medaka estrogen receptor (medERα), human constitutive androstane receptor (CAR), and human aryl hydrocarbon receptor (AhR) and the coactivator TIF2, in accordance with the method of Nishikawa et al. (1999). Expression plasmids for the hormone receptor ligand binding domain and pGAAD24-TIF-2 were introduced into yeast cells that carried the β-galactosidase reporter gene (Nishikawa et al., 1999)[1]. The assays adopted the chemiluminescent reporter gene (for β-galactosidase) method employing a 96-well culture plate (Shiraishi et al., 2000, )[4]. Yeast cells were preincubated for 24 h at 30 °C with shaking in modified SD medium (lacking tryptophan and leucine, 0.86% dextrose) and the cell density was adjusted to an absorbance of 1.75–1.85 at 595 nm. The medium (60 μl) was placed in the wells of the first row of a black 96-well culture plate for chemiluminescence measurement. Wells in rows 2–8 were charged with a solution of 2% DMSO in the medium (60 μl). A solution of test compound (1 mM in DMSO, 20 μl) was added to the medium (480 μl) and aliquots of this mixture (60 μl) were also added to the wells of the first row of the plate. The test solution was serially diluted from row 1 to 7 (each 2×) and then the yeast cell suspension (60 μl) was also added to each well (including those in row 8, which served as the blank control). Thus, the first row contained a 10 μΜ solution of the test chemical, the second row a 5 μM solution, and so on. After the addition of the yeast suspension and vortex mixing, the plates were incubated at 30 °C under high humidity for 4 h. A solution (80 μl) for inducing chemiluminescence from released β-galactosidase, consisting of reaction buffer (30 μl) containing GalactLux substrate (AURORA GAL-XE, ICN Biomedicals, Inc., Irvine, CA) and zymolylase 20T solution for enzymatic digestion (50 μl), was added to each well. The plate was incubated at 37 °C for 1 h and then placed in a 96-well plate luminometer (Luminescencer JNR AB2100, Atto Corp., Tokyo, Japan) and a light emission accelerator solution (AURORA GAL-XE 50 μl) was added to each well using the luminometer pump. The chemiluminescence produced by β-galactosidase in each well was measured.

A “positive” result in the antagonist activity test was judged to have occurred when the maximum suppression of the chemiluminescence of β-galactosidase activity toward E2 was more than 40% lower than that for the solvent control and when dose dependence was observed over at least three consecutive points. For all substances judged to be positive, the corresponding IC50 was calculated. The quality control of the ER- and AR-antagonism tests was performed by defining the concentration range of IC50 in the positive control and retesting if results outside that range were obtained. Each positive control was 17β-estradiol (The highest concentration in test well medium was 4 nM, and 7 stages were set with double dilution. The following values are same.): hER-, medER-agonistic), β-naphthoflavone (10 nM: AhR-agonistic, with 600–2000 pM of E2), 4-hydroxy-tamoxygen (50 nM: hER-antagonistic, with 20 nM of hydroxyltestosterone), and 1-nitropyrene (50 nM: AR-antagonistic).

The allowable range was as follows: ER-antagonist test, IC50 of 4-hydroxy-tamoxygen was 1200–1800 nM; AhR-antagonist test, IC50 of 1-nitropyrene was 100–500 nM. The YTOX test was also conducted separately to clarify that the decrease in luminescence was due not to toxicity of the test chemicals to the yeast but to antagonism.
