# Supplementary material for: Screening data for the endocrine disrupting activities of 583 chemicals using the yeast two-hybrid assay
Source: Data Brief. 2018 Nov 23;21:2543–6. doi: 10.1016/j.dib.2018.11.071 (PMC6288393; doi:10.1016/j.dib.2018.11.071)
Supplement: Supplementary file 1 — Supplementary material [file mmc1.doc]

Conflict of Interest and Authorship Conformation Form

Please check the following as appropriate:

- All authors have participated in (a) conception and design, or analysis and interpretation of the data; (b) drafting the article or revising it critically for important intellectual content; and (c) approval of the final version.
- This manuscript has not been submitted to, nor is under review at, another journal or other publishing venue.
- The authors have no affiliation with any organization with a direct or indirect financial interest in the subject matter discussed in the manuscript
- The following authors have affiliations with organizations with direct or indirect financial interest in the subject matter discussed in the manuscript:

Author’s name Affiliation

Fujio Shiraishi National Institute for Environmental Studies

Ryo Kamata Kitasato University

Masanori Terasaki Iwate University

Hidetaka Takigami National Institute for Environmental Studies

Yoshitaka Imaizumi National Institute for Environmental Studies

Mayuko Yagishita National Institute for Environmental Studies

Daisuke Nakajima National Institute for Environmental Studies
